# Molecular Detection of Adenoviruses, Rhabdoviruses, and Paramyxoviruses in Bats from Kenya

**DOI:** 10.4269/ajtmh.13-0664

**Published:** 2014-08-06

**Authors:** Christina Conrardy, Ying Tao, Ivan V. Kuzmin, Michael Niezgoda, Bernard Agwanda, Robert F. Breiman, Larry J. Anderson, Charles E. Rupprecht, Suxiang Tong

**Affiliations:** Division of Viral Disease, Centers for Disease Control and Prevention, Atlanta, Georgia; National Center for Emerging and Zoonotic and Infectious Diseases, Centers for Disease Control and Prevention, Atlanta, Georgia; The Global Alliance for Rabies Control, Manhattan, Kansas; Zoology Department, National Museum, Nairobi, Kenya; Centers for Disease Control and Prevention–Kenya, Nairobi, Kenya; Emory University, Atlanta, Georgia

## Abstract

We screened 217 bats of at least 20 species from 17 locations in Kenya during July and August of 2006 for the presence of adenovirus, rhabdovirus, and paramyxovirus nucleic acids using generic reverse transcription polymerase chain reaction (RT-PCR) and PCR assays. Of 217 bat fecal swabs examined, 4 bats were adenovirus DNA-positive, 11 bats were paramyxovirus RNA-positive, and 2 bats were rhabdovirus RNA-positive. Three bats were coinfected by two different viruses. By sequence comparison and phylogenetic analysis, the Kenya bat paramyxoviruses and rhabdoviruses from this study may represent novel viral lineages within their respective families; the Kenya bat adenoviruses could not be confirmed as novel, because the same region sequences from other known bat adenovirus genomes for comparison were lacking. Our study adds to previous evidence that bats carry diverse, potentially zoonotic viruses and may be coinfected with more than one virus.

## Introduction

Over one-half of all known human pathogens originated from animals, and over 75% of emerging infectious diseases identified in the last three decades were zoonotic.^1^ The threat of veterinary pathogens to human health continues to grow because of increasing population density and urbanization, global movement of people and animals, and deforestation accompanied by increased proximity of human and wildlife habitats. Recent emerging infectious diseases have been concentrated in tropical Africa, Latin America, and Asia, with outbreaks usually occurring within populations living near wild animals.^1^ Identification of animal reservoirs from which zoonosis may emerge and detection and characterization of pathogens in these reservoirs will facilitate timely implementation of control strategies for new zoonotic infections.^2^ Therefore, pathogen discovery studies in animal reservoirs represent an integral part of public health surveillance.

Bats have long been known as natural hosts for lyssaviruses, and more recently, they have been recognized as potential reservoirs for emerging human pathogens, including henipaviruses, filoviruses, and severe acute respiratory syndrome (SARS) related coronaviruses.^3^,^4^ Novel viruses are documented in bats every year, which has drawn increasing attention to these mammalian reservoirs that are uniquely associated with a variety of known and potential zoonotic pathogens. In this study, we report the detection of nucleic acids of adenoviruses, rhabdoviruses, and paramyxoviruses in bats from Kenya.

## Study

Field sampling of bats was implemented in Kenya for zoonotic surveillance within the framework of the Global Disease Detection Program of the Centers for Disease Control and Prevention. Detailed information on bat capture and handling is described elsewhere.^5^ In this study, fecal swabs (*N* = 217) collected during July and August of 2006 from apparently healthy bats representing 21 species in 13 genera from 17 locations within Kenya were screened for the presence of adenovirus, polyomavirus, rhabdovirus, and paramyxovirus nucleic acids using generic reverse transcription polymerase chain reaction (RT-PCR) and PCR assays.^6^–^8^ Polyomavirus detection has been described previously.^9^ Positive PCR products were purified, sequenced, and analyzed on an ABI Prism 3130 Automated Sequencer (Applied Biosystems, Foster City, CA). Sequences were aligned with those sequences of known representatives of the same viral families, and phylogenetic reconstructions were performed using Bayesian Markov Chain Monte Carlo analysis implemented in BEAST Program under the Hasegawa-Kishino-Yano (HKY) substitution model.^10^

Of 217 fecal swabs tested (Table 1), adenovirus DNA was detected in 4 samples from *Chaerephon* sp. (*N* = 2) and *Otomops martiensseni* (*N* = 2); paramyxovirus RNA was detected in 11 samples from *Cardioderma cor* (*N* = 1), *Chaerephon* sp. (*N* = 1), *O. martiensseni* (*N* = 5), *Rousettus aegyptiacus* (*N* = 2), *Miniopterus minor* (*N* = 1), and *M. natalensis* (*N* = 1); and rhabdovirus RNA was detected in 2 samples from *Chaerephon* sp. (*N* = 1) and *M. africanus* (*N* = 1). Three bats harbored viruses from two different viral families. One *O. martiensseni* bat was coinfected with a paramyxovirus and a polyomavirus previously described.^9^ Another *O. martiensseni* bat was coinfected with an adenovirus and a paramyxovirus. One *Chaerephon* sp. bat was coinfected with a rhabdovirus and a polyomavirus.^9^ Additional specimens of lung, kidney, liver, and/or brain tissues from nine bats that had paramyxovirus RNA-positive fecal swabs were also tested for paramyxovirus RNA. Four bats (KY149, KY151, KY166, and KY291) tested positive on kidney tissues, and one bat (KY159) tested positive on kidney, lung, and liver tissues. The KY159 bat kidney and lung tissues were coinfected with two different types of paramyxoviruses. One sequence was the same as identified in the fecal swab (KY159a), and the other sequence represented a rubula-related virus (KY159b). These findings support an assumption for an active viral infection rather than simple transit of ingested infected material through the digestive tract of the bat. In addition, positive identification of paramyxovirus RNA in these tissues may stem from infection at these sites or possible viremia. Attempts to propagate paramyxovirus-positive, adenovirus-positive, and rhabdovirus-positive tissue samples in cell culture have been unsuccessful to date. Genome sequencing of paramyxovirus-positive rectal samples by 454 pyrosequencing failed to amplify paramyxovirus sequences, probably because of insufficient load of viral RNA in the sample.

**Figure 1.**
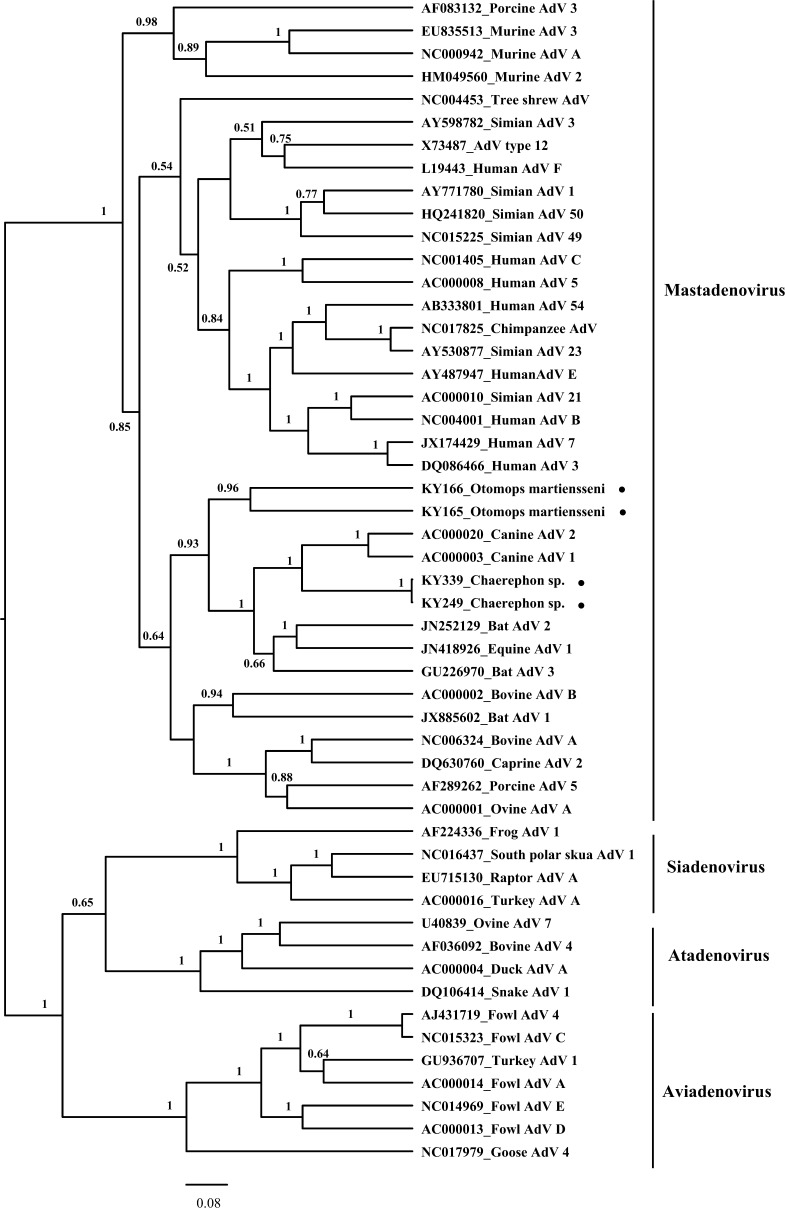
Phylogenetic analysis of adenoviruses. The adenovirus phylogenetic tree was generated using the partial hexon gene sequences (630 nucleotides) from a representative sample comprising 47 known adenoviruses. Viral sequences identified in bats from Kenya are noted by black circles. The trees are drawn to scale, with branch lengths measured in the number of substitutions per site. The Bayesian posterior probabilities (> 0.5) are shown at nodes.

Phylogenetic analysis of four Kenya bat adenoviruses based on partial hexon gene sequences (630 base pairs) and representative sequences comprising 47 known adenoviruses (Figure 1) showed that these viruses grouped together within the *Mastadenovirus* genus and were most closely related to canine adenovirus types 1 and 2 and bat adenoviruses from China (bat adenovirus 3 from *Myotis ricketti*) and Germany (bat adenovirus 2 from *Pipistrellus pipistrellus*).^11^,^12^ Interestingly, many other bat mastadenoviruses recently identified from China, Hungary, India, Japan, North America, Brazil, and Spain were also shown to be distantly related to canine adenovirus types 1 and 2 based on a different region of DNA polymerase sequences.^12^–^17^ These data may suggest that canine adenoviruses 1 and 2 and the bat adenoviruses share a common ancestor. The KY249 and KY339 sequences from *Chaerephon* sp. bats were nearly identical (99% nucleotide identity) but distinct from KY165 and KY166 sequences obtained from *O. martiensseni* bats (61–63% nucleotide identity). The KY165 and KY166 sequences shared only 59% nucleotide identity with each other. These four Kenya bat adenovirus sequences share 58–66% nucleotide identity with their most closely related adenoviruses: bat adenoviruses 2 and 3.

**Figure 2.**
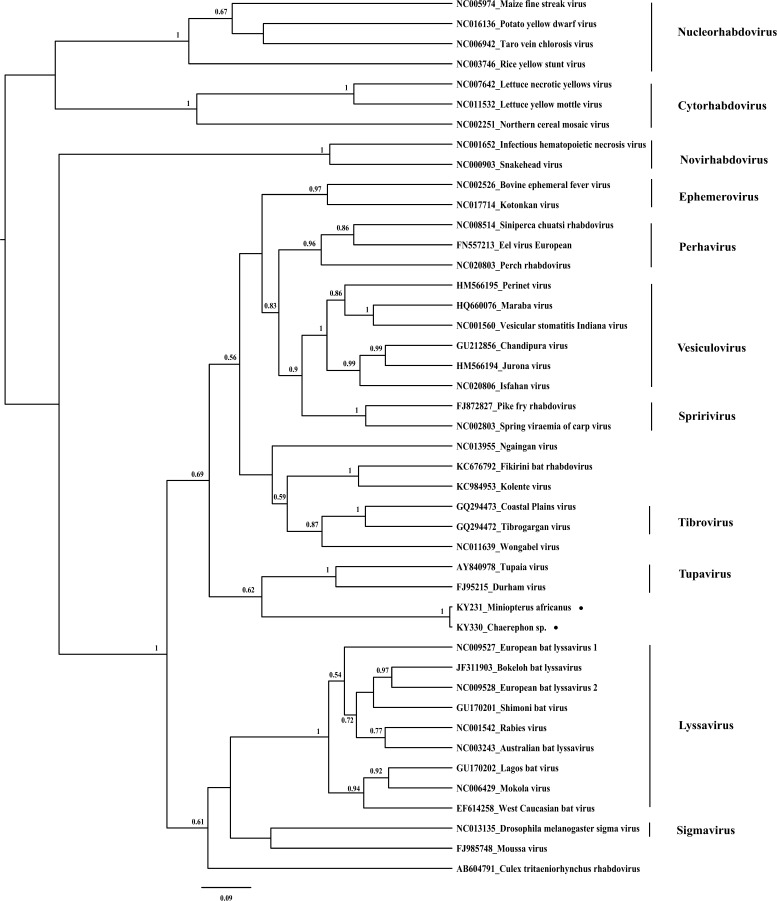
Phylogenetic analysis of rhabdoviruses identified in bats from Kenya. The rhabdovirus phylogenetic tree was generated based on partial RDRP gene sequences (219 nucleotides) from representative known rhabdoviruses. Viral sequences identified in bats from Kenya are noted by black circles. The trees are drawn to scale, with branch lengths measured in the number of substitutions per site. The Bayesian posterior probabilities (> 0.5) are shown at nodes.

Two nearly identical rhabdovirus partial RDRP gene sequences (KY231 and KY330; 219 nucleotides) were identified in bats from two different species (*M. africanus* and *Chaerephon* sp.) in different locations. They were divergent from their near neighbors (about 60–65%% nucleotide identity by BLAST), for which relevant genetic information (L gene sequences overlapping our PCR product) is available in GenBank for comparison (Figure 2).

**Figure 3.**
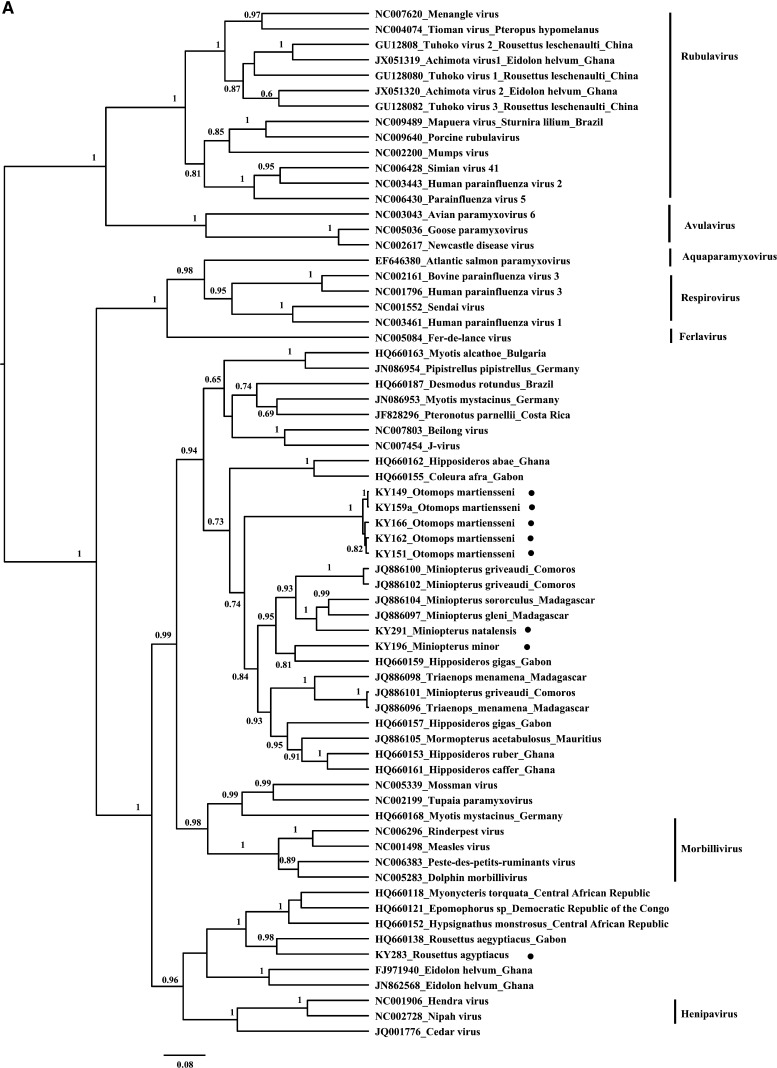

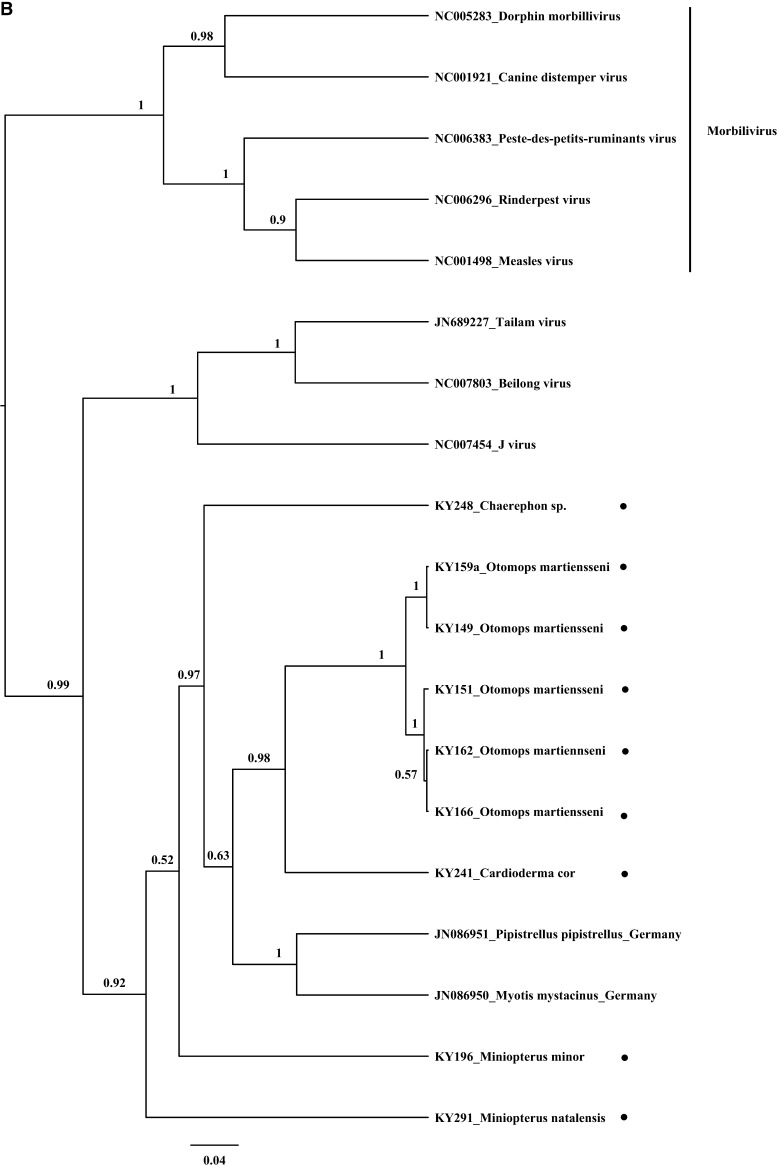

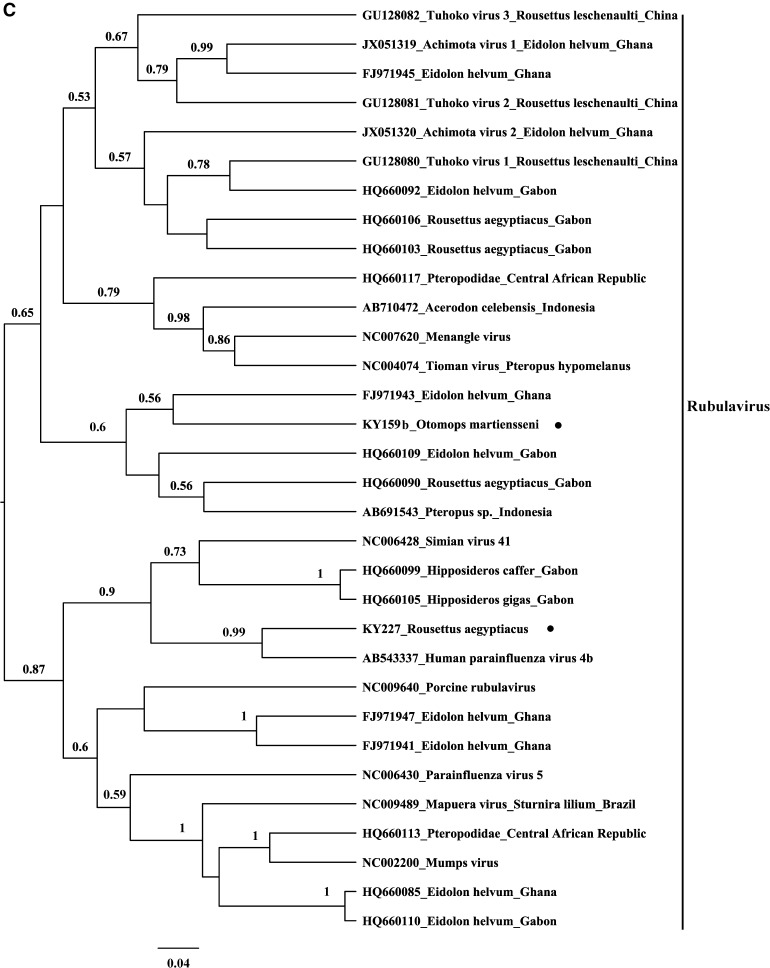
Phylogenetic analysis of paramyxoviruses identified in bats from Kenya. The paramyxoviruses phylogenetic trees were generated based on (**A**) amplicon sequences (305 nucleotides) from generic RT-PCR assays of the subgroup *Respirovirus-Morbillivirus-Henipavirus*, (**B**) amplicon sequences (294 nucleotides) from generic RT-PCR assays of the *Paramyxovirinae*, and (**C**) amplicon sequences (216 nucleotides) from generic RT-PCR assays of the subgroup *Avulavirus-Rubulavirus* from representative known paramyxovirues. Viral sequences identified in bats from Kenya are noted by black circles. The trees are drawn to scale, with branch lengths measured in the number of substitutions per site. The Bayesian posterior probabilities (> 0.5) are shown at nodes.

Twelve paramyxovirus sequences were detected from 11 Kenya bats by three different generic paramyxovirus RT-PCR assays as described before.^7^ The KY159 bat was identified as having two different paramyxovirus sequences (KY159a and KY159b). Seven sequences (KY196, KY291, KY149, KY151, KY159a, KY162, and KY166) were detected by both generic RT-PCR assays of the subgroup of *Respirovirus-Morbillivirus-Henipavirus* (Figure 3A) and *Paramyxovirinae* (Figure 3B), and five sequences were detected by generic RT-PCR assays of *Paramyxovirinae* (KY241 and KY248) subgroup of either *Respirovirus-Morbillivirus-Henipavirus* (KY283) or *Avulavirus-Rubulavirus* (KY227 and KY159b) (Figure 3C). These 12 paramyxovirus sequences belonged to three different taxa groups based on partial polymerase gene (L) sequences. Nine sequences were grouped with Beilong virus, J virus, and unclassified bat paramyxoviruses within *Paramyxovirinae* that were identified in bats from Central and South America, Indian Ocean islands, Africa, and Europe.^18^–^22^ Notably, these nine sequences formed five distinct lineages (KY196, KY291, KY241, KY248, KY149-KY151-KY159a-KY162-KY166) with 72–78% nucleotide identity between each lineage, suggesting five novel lineages. Sequences within the lineage KY149-KY151-KY159a-KY162-KY166 shared 97–100% nucleotide identity with each other. Interestingly, KY196 and KY291 sequences from the Kenya *Miniopterus* bats were grouped with sequences of paramyxoviruses from other insectivorous bats, including *Miniopterus* originating from Comoros and Madagascar and a *Hipposideros gigas* bat from Gabon, respectively (Figure 3A).^19^,^22^ One sequence (KY283) identified in *R. aegyptiacus* was grouped with another known bat henipavirus-related virus identified in a *R. aegyptiacus* bat from Gabon (72% nucleotide identity). This finding supports the previous suggestion that pteropodid bats maintain circulation of henipavirus-related viruses in several continents of the Old World.^22^ Two sequences (KY227 and KY159b) identified in an *R. aegyptiacus* bat and an *O. martiensseni* bat, respectively, were grouped within the *Rubulavirus* genus (Figure 3C). KY227 was more related to human parainfluenza virus 4b (HPIV4b; 85% nucleotide identity), and KY159b from the *O. martiensseni* bat was more related to GH1a from the *Eidolon helvum* bat from Ghana (65% nucleotide identity) based on partial L gene sequences. We detected paramyxovirus RNA from three insectivorous bat genera (*Cardioderma*, *Chaerephon*, and *Otomops*) that were not previously reported to be carriers of paramyxoviruses.

## Conclusion

We detected distinct viral DNA and RNA from the families *Adenoviridae*, *Rhabdoviridae*, and *Paramyxoviridae* in Kenya bats using generic family and/or genus RT-PCR and PCR assays. Although the limited length of genome sequences and the low Bayesian posterior probabilities do not provide reliable phylogenetic comparisons and taxonomic inferences, the magnitude of the genetic distance (85% or less nucleotide identity in highly conserved genomic regions) between the Kenya bat paramyxoviruses and rhabdoviruses from this study and other known paramyxoviruses and rhabdoviruses might be suggestive of their being novel viral lineages within their respective families. The Kenya bat adenoviruses could not be confirmed as novel, because many bat adenoviruses have recently been described that are also related to canine adenovirus types 1 and 2, and we were unable to obtain sequences from the same region of the genome for direct comparison.

Our findings also show that Kenya bats maintain as much genetic diversity in paramyxoviruses as bats in other geographic locations. The concurrent detection of both RNA and DNA viruses in apparently healthy bats supports evidence that bats may be carriers of more than one virus. Of note, many bats that tested positive for adenovirus, paramyxovirus, and polyomavirus were *O. martiensseni* from Suswa Cave. Suswa Cave houses one of the largest known colonies of *O*. *martiensseni* and has an extensive history of guano mining and tourist visits.^23^ Anthropogenic activities, including guano mining, cave tourism, hunting, and consumption of bats, likely increase the chance of zoonotic infection spillovers from these bats.^2^ Studying viral diversity in bats and their biology will help understanding and response to novel emerging viruses.

## Figures and Tables

**Table 1:** Positive PCR results per bat species and geographical locations

Bat species/location	Number of bats tested	Adenovirus	Paramyxovirus	Rhabdovirus	Polyomavirus*
*Cardioderma cor*					
Kisumu	1				1
Panga Yambo cave	10		1		
Tsavolite goldmine	3				
*Chaerephon* sp.					
Kisumu	13	1		1	5
Moi University	16	1	1		2
Asembo Bay	6				
*Chaerephon pumilus*					
Marungu	5				
Shimoni cave	1				
*Coleura afra*					
Marungu	1				
Shimoni cave	1				
*Eidolon helvum*					
Vihiga District	9				1
*Epomophorus wahlbergi*					
Nairobi	3				
*Hipposideros commersoni*					
Shimoni cave	9				1
*Hipposideros ruber*					
Kakamega cave	2				
Makingeny cave	4				
*Lissonycteris angolensis*					
Kakamega cave	11				1
Kisumu	1				1
*Miniopterus africanus*					
Chyulu National Park	9			1	1
*Miniopterus inflatus*					
Kakamega cave	10				
*Miniopterus natalensis*					
Kitum cave	8		1		
*Miniopterus minor*					
Three caves	16		1		
*Otomops martiensseni*					
Suswa cave	19	2	5		6
*Pipistrellus* sp.					
Nairobi	1				
Kisumu	4				
*Rhinolophus* sp.					
Three caves	1				
Shimoni cave	1				
No information	2				
*Rhinolophus clivosus*					
Nairobi	5				
*Rhinolophus hildebrandtii*					
Chyulu National Park	4				
*Rousettus aegyptiacus*					
Three caves	11				3
Kitum cave	10		1		
Makingeny cave	9				
Watamu cave	6		1		1
*Taphozous nudiventris*					
Marungu	2				
*Taphozous hildegarde*					
Shimoni cave	3				
Total	217	4	11	2	23

* See ref. 9.
